# Cluster-Randomized Controlled Study of SMS Text Messages for Prevention of Mother-to-Child Transmission of HIV in Rural Kenya

**DOI:** 10.1155/2016/1289328

**Published:** 2016-12-08

**Authors:** Seble G. Kassaye, John Ong'ech, Martin Sirengo, Judith Kose, Lucy Matu, Peter McOdida, Rogers Simiyu, Titus Syengo, David Muthama, Rhoderick Machekano

**Affiliations:** ^1^Department of Medicine, Georgetown University, Washington, DC, USA; ^2^Kenyatta National Hospital, Nairobi, Kenya; ^3^Kenya National AIDS & STI Control Programme, Nairobi, Kenya; ^4^Elizabeth Glaser Pediatric AIDS Foundation, Nairobi, Kenya; ^5^Elizabeth Glaser Pediatric AIDS Foundation, Washington, DC, USA

## Abstract

*Background*. Antiretroviral medications are key for prevention of mother-to-child transmission (PMTCT) of HIV, and transmission mitigation is affected by service delivery, adherence, and retention.* Methods*. We conducted a cluster-randomized controlled study in 26 facilities in Nyanza, Kenya, to determine the efficacy of SMS text messages on PMTCT outcomes. The relative risk and confidence intervals were estimated at the facility level using STATA.* Results*. 550 women were enrolled, from June 2012 to July 2013. The median age was 25.6 years, and 85.3% received ARVs. Maternal ARV use was similar between the intervention and control arms: 254/261 (97.3%) versus 241/242 (99.6%) at 34–36 weeks of gestation and 234/247 (94.7%) versus 229/229 (100%) at delivery. Among infants, 199/246 (80.9%) and 209/232 (90.1%) received ARVs (RR: 0.91; 95% CI: 0.77–1.14); 88% versus 88.6% were tested for HIV at 6 weeks, with 1/243 (0.4%) and 3/217 (1.4%) positive results in the intervention and control arms, respectively. Communication increased in both the intervention and control arms, with the mean number of 7.5 (SD: 5.70) compared with 6 (SD: 9.96), *p* < 0.0001.* Conclusions*. We identified high ARV uptake and infant HIV testing, with very low HIV transmission. Increased communication may influence health-seeking behaviors irrespective of technology. The long-term effectiveness of facilitated communication on PMTCT outcomes needs to be tested. The study has been registered on ClinicalTrials.gov under the identifier NCT01645865.

## 1. Introduction

HIV remains a significant cause of morbidity and mortality among children worldwide, with the primary mode of transmission being through vertical transmission of HIV from mothers to infants during pregnancy, delivery, and breastfeeding [1]. Use of antiretroviral medications, elective cesarean section among women with persistent viremia, and formula replacement feeding have resulted in <2% HIV transmission among HIV-exposed infants in developed countries [2]. Clinical studies performed in sub-Saharan Africa using increasingly complex regimens resulted in significant decreases in perinatal HIV transmission, ranging within 1.7–6% [3–5]. However, an estimated 170,000 children acquired HIV infection in 2014, remaining above the target of decreasing HIV transmission to less than 5% [1, 6]. Over 90% of these infections were in sub-Saharan Africa where limited access to and usage of health services promotes vertical transmission of HIV.

There is increased interest in the use of technology-based methods to improve health service delivery. Even among women who attend antenatal clinics, uptake of key services including initiation of antiretroviral therapy is often suboptimal. At the time this study was planned in 2011, approximately 71% of pregnant women were tested for HIV in Kenya; 62% of HIV-positive pregnant women received antiretrovirals for prophylaxis or treatment (excluding single dose nevirapine); and 44% of women delivered in health facilities; 63% of HIV-exposed infants received prophylactic antiretrovirals and 40% were tested for HIV at 6–8 weeks of age [7, 8]. Little information is available on adherence rates to ART or infant prophylaxis during breastfeeding.

Mobile health (mHealth) interventions have been shown to improve adherence to ART among adults and improve health service usage [9, 10], but support for this approach during pregnancy is limited. mHealth interventions during pregnancy were found to increase postpartum retention, although the observed increase was lower than expected based on regional data [11].

As there is wide access to mobile phones in Kenya [12], the Elizabeth Glaser Pediatric AIDS Foundation, in collaboration with the Ministry of Health in Kenya, designed an intervention study to determine the utility of short message service (SMS) text messages to improve uptake of antenatal and prevention of MTCT (PMTCT) services. We conducted a cluster-randomized study in Nyanza province in government supported clinical sites to determine whether SMS text messages would influence uptake of essential PMTCT-related health services by improving communication between women and their health providers in Nyanza province, Kenya, a rural setting with 15% HIV prevalence [13] (ClinicalTrials.gov Identifier: NCT01645865).

## 2. Methods

This study was designed to test the hypothesis that reinforcement of key messages delivered via SMS would result in improvement in completion of key PMTCT cascade milestones. SMS text messages were designed based on our formative research conducted among HIV-positive women in the PMTCT program and community health workers [14]. Primary outcomes were antiretroviral uptake at 34–36 weeks of gestation and at delivery. Secondary outcomes included facility delivery, infant's uptake of HIV prophylaxis, infant HIV testing at 6–8 weeks after delivery, and HIV transmission.

### 2.1. PMTCT Program Description

Women identified with HIV infection during antenatal care (ANC) receive either antiretroviral prophylaxis with zidovudine (AZT) starting in the second trimester of pregnancy or combination antiretroviral therapy (ART), with a CD4+ T-lymphocyte count less than 350 per cu mm used to determine the need for ART [15]. During the course of this study, guidelines evolved to expand HIV treatment to all HIV-positive pregnant women irrespective of CD4+ T-lymphocyte count, and this guidance was implemented in the high volume facilities included in this study [16]. All infants receive nevirapine prophylaxis from birth until 6 weeks postpartum, extended throughout breastfeeding if the mother is not receiving ART. Infants are tested for HIV using DNA PCR testing at 6 weeks postpartum.

Community health workers (CHWs) serve as health extension workers in PMTCT programs in rural Kenya and are integral members of the communities in which they serve. CHWs ensure that women follow up in ANC, provide ongoing pregnancy and newborn health education to women, and encourage adherence to antiretrovirals.

### 2.2. Site Selection and Randomization

In this cluster-randomized study, 26 of the 54 health facilities within Homa Bay and Rachuonyo districts in Nyanza province were selected. Ten facilities serving <20 HIV-positive pregnant women yearly were excluded due to anticipated low enrollment. A volume-stratified sampling method was applied to ensure inclusion of a representative sample of types of health facilities among the remaining facilities. Two hospitals in Homa Bay were selected, and two of the four hospitals in Rachuonyo district were randomly selected and included. Eight health centers and fourteen dispensaries were randomly selected from the remaining forty health facilities. All health facilities were randomly allocated to be an intervention or control site, stratified by high volume (hospitals) and medium and low volumes (health centers and dispensaries).

### 2.3. Study Population

Consecutive eligible HIV-positive pregnant women presenting for ANC were invited to participate. Women were eligible to enroll in the study if they were less than 32 weeks of gestational age, were not currently receiving antiretroviral therapy, were planning to remain in the area for the duration of the study period, and agreed to follow-up of their infants until 6 weeks following delivery. Only women in the intervention arm were required to have access to a mobile phone (individual or shared) and be willing to receive SMS text messages. Male partners of women were also permitted to enroll in the study.

### 2.4. Study Intervention

An mHealth platform was designed and built in collaboration with DewCIS Solutions Ltd., a Kenya-based Information Technology company. A mobile web-based communications software system allowed for semiautomated delivery of preloaded messages. Participants were allocated to list-serves based on pregnancy stage to ensure delivery of appropriate messages. Participants were able to communicate with their assigned CHW by phone or text message to ask questions, report concerns, or provide notice of key transition points such as onset of labor or delivery. Participants were provided with airtime, equivalent of $3 US dollars, at each study visit to cover communication costs. Community health workers in both the intervention and control sites were retrained on PMTCT service delivery and on appropriate messaging to women during pregnancy and postpartum. Community health workers were provided with the equivalent of $6 US dollars for airtime expenses.

### 2.5. SMS Text Messages

SMS text messages were designed by the study team to address areas of need identified during our formative research [14]. SMS text messages were iteratively edited for content and tone and translated in collaboration with local study team members fluent in the local language. Participants received 3 to 6 SMS messages each week at a self-selected time of day and in their preferred language. The thematic areas covered four broad categories: PMTCT services, including appointment reminders and adherence support; motivational messages of hope and encouragement; male-partner involvement and engagement in delivery planning; and essential maternal child health messages including warning signs and nutrition.

### 2.6. Study Visits

Women underwent structured interviews at four visits (enrollment; 36 weeks of gestation; delivery, 7 days postpartum; and 6–8 weeks postpartum) to record self-reported adherence to antiretrovirals in the past week, number and mode of communication between the participant and health workers, time and place of delivery, infant feeding practices, and any intervening clinical outcomes.

### 2.7. Sample Size and Power Calculations

The sample size for the study was estimated by inflating the sample size from a 2-arm simple randomized study using the design effect (DE), 1 + (*n* − 1)*ρ*, where *n* is the cluster sample size and *ρ* is the intracluster correlation. As there were no published data on the intracluster correlation coefficients for the selected outcomes, we estimated the coefficient of variation for a number of plausible intracluster correlation coefficients [17]. With a sample size of 286 women from 13 clusters per study arm, assuming an intracluster correlation <0.05 (range 0.01–0.51), the study had 80% power to detect an absolute effect size of 20% between intervention and control sites for the selected outcomes, including uptake of antiretrovirals, facility-based deliveries, and HIV infant testing at 6 weeks of age, after factoring in possible loss to follow-up of 10%.

### 2.8. Statistical Methods

Baseline demographics and clinical characteristics were summarized using proportions for categorical variables and medians with associated interquartile ranges for continuous variables, stratified by intervention group. All outcomes were summarized at the individual and cluster level. For the individual level analysis, the overall proportion of women with the outcome of interest was estimated by intervention assignment. In the cluster level analysis, the proportion of women with the outcome of interest was estimated for each health facility. The study arm proportion was obtained as the mean of facility level proportions together with the standard deviation of the facility means.

In our study, the intervention was randomly allocated to health facilities; hence, it is the unit of analysis. The effect of the mHealth intervention on each of the outcomes was estimated by comparing the two sets of health facility-specific proportions using two-sample *t*-tests. Differences between group proportions and associated confidence levels were estimated using *t*-distribution. Statistical significance tests were confirmed using the nonparametric Wilcoxon rank-sum tests. Since the number of health facilities per group was considered small, the comparison of facility level summaries was conducted using *t*-tests as this has been demonstrated to be the most robust approach compared to regression methods. The effect of the intervention was also estimated using relative risks and associated confidence intervals.

### 2.9. Adjusted Effect of the Intervention

We used a two-stage analytic approach to estimating the adjusted effect of the intervention. In the first stage, we estimated cluster level residuals for each health facility. For each outcome, we fitted a logistic regression model of the outcome and potential confounding variables including participant age, gestational age, whether the woman was newly diagnosed with HIV, and disclosure of HIV status to her partner and family. To measure the intervention effect as a risk difference, we estimated the residual for each health facility by taking the difference of the number of observed outcome events and the number of expected outcomes events. The adjusted risk difference was estimated by the difference between the mean facility-specific residuals in the intervention arm and the mean facility-specific residuals in the control. For the relative risk effect measure, the cluster-specific residual was estimated by the ratio of the observed outcome events to the expected outcome events for each health facility. The adjusted relative risk is estimated by ratio of the mean of the ratio residuals for clusters in the mHealth intervention arm to the mean of the ratio residuals for clusters in the control arm. Associated confidence intervals for each effect measure were also estimated.

### 2.10. Ethical Considerations

The protocol was approved by the Kenyatta National Hospital/University of Nairobi (KNH/UON) ethics review committee, the World Health Organization Institutional Review Board (IRB), and the Georgetown University IRB recognized KNH/UON as the IRB of record. All participants provided written informed consent.

## 3. Results

**Figure 1:**
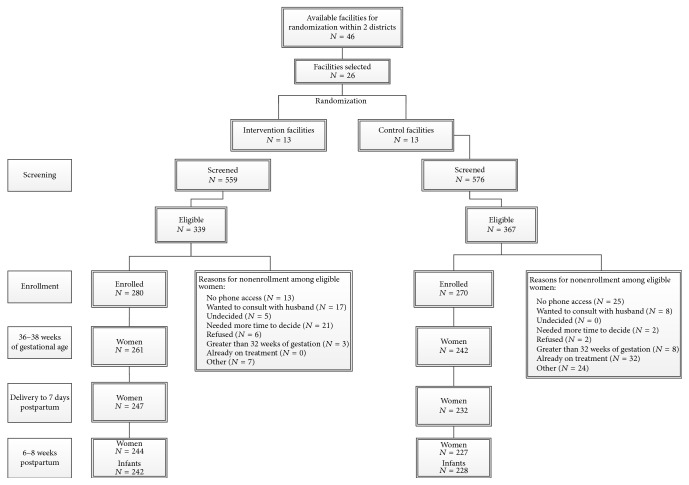
Site randomization and participant flow from screening and enrollment and through follow-up.

A total of 550 women were enrolled into the study from June 2012 to June 2013, 280 in the intervention arm and 270 in the control arm (Figure 1). Women in both groups were similar, with a median age of 25.6 years and mostly primary level education (Table 1). However, more women were newly diagnosed with HIV during this pregnancy in the control arm (65.6%) compared with in the intervention arm (55.4%). Only 57/550 (10.4%) of women had baseline CD4+ T-lymphocyte testing, with overall median CD4+ T-lymphocyte count of 486 per cu mm.

### 3.1. Maternal Uptake of Antiretroviral Medications

Uptake of antiretrovirals was high in both the intervention and control arms (Table 2). Antiretrovirals were given to 86.8% and 83.7% of women in ANC in the intervention and control arms, respectively, of whom 98.4% reported taking their antiretroviral medications at 34–36 weeks with no significant difference between arms (adjusted relative risk (aRR) 1.04, 95% CI: 0.91–1.18). Among women in the intervention arm, 7.8% reported missing at least one dose of their antiretrovirals in the preceding week compared to 5.4% in the control arm (aRR 1.25, 95% CI: 0.43–3.60).

At delivery, 94.7% of women in the intervention arm reported taking their antiretroviral medications compared to 100% in the control arm (aRR 1.01, 95% CI: 0.88–1.16), with 22.9% on ART and 6.3% of women reporting missing at least one dose of their antiretroviral medication in the past week. 21% of women continued on ART at 6–8 weeks postpartum, similar to the intervention (20.1%) and control arm (21.2%) (aRR 1.01, 95% CI: 0.29–3.45).

### 3.2. Facility-Based Delivery and Infant Uptake of Antiretrovirals

Only 55% of women delivered in a health facility, 50.6% in the intervention arm and 59.5% in the control arm. 85.4% of infants were reported to have started taking antiretroviral prophylaxis at delivery, 80.9% in the intervention arm and 90.1% in the control arm (aRR: 0.91; CI 0.77–1.14). Infant antiretroviral receipt increased by 6 weeks postpartum to 98.9%, 99.6% in the intervention arm and 97.8% in the control arm.

### 3.3. Infant HIV Testing and HIV Transmission

Infant HIV testing at 6 weeks of age was 88.3% overall, with 88% in the intervention arm and 88.6% in the control arm. Overall HIV transmission was 0.9%: one infant in the intervention arm (0.4%) and three infants (1.4%) in the control arm had a positive HIV DNA PCR test at 6 weeks postpartum and this was not significantly different between groups.

### 3.4. Communication between the Participant and CHWs

**Figure 2:**
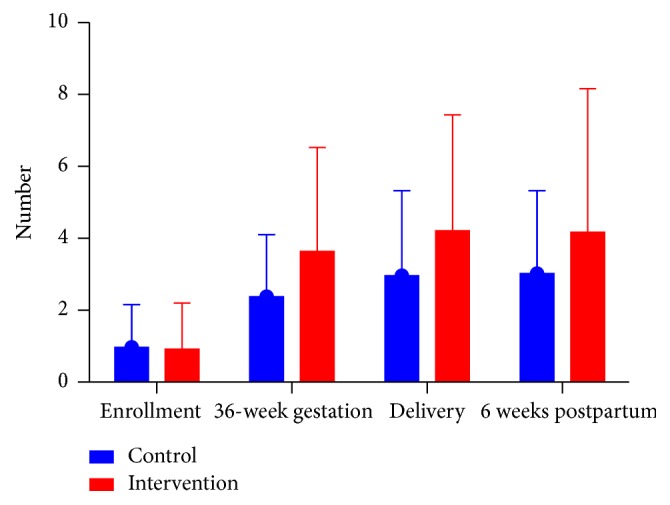
Frequency of communication from enrollment until 6 weeks postpartum. Depicting the mean frequency and standard deviation of communication between women and health care workers as reported by women at enrollment, between enrollment and 36 weeks of gestation, between 36 weeks of gestation and within 7 days of delivery, and between the delivery visit and 6–8 weeks postpartum.

At baseline, the mean number of communications both face-to-face and via mobile phone between participants and health workers was similar between groups, 0.92 and 0.96 in the intervention and control sites, respectively (Figure 2). Communication increased in both groups during subsequent visits, with greater cumulative increased communication by delivery in the intervention than control arm, 7.5 (SD: 5.70) compared with 6 (SD: 9.96), *p* < 0.0001 (two-sample Wilcoxon rank-sum test). Communication via mobile phones were higher in the intervention than in the control sites, with a median of 3 compared with 1 conversation among women and CHWs. There was no difference in the number of face-to-face conversations between intervention and control groups, with a median of 5 conversations per group.

### 3.5. Communication and Infant HIV Testing

There was higher cumulative communication among women whose infants were not tested for HIV, 11.5 (SD: 10.2), compared with 9 (SD: 7.11) communications among those tested for HIV, *p* = 0.0176 (two-sample Wilcoxon rank-sum test). Higher frequency of communication was associated with a lower odds of infant HIV testing both unadjusted (OR: 0.92; 95% CI: 0.87–0.99) and adjusted for age, number of pregnancies, gestational age at first ANC visit, education, disclosure of HIV status to partner, and disclosure of HIV status to family (OR: 0.91; 95% CI: 0.87–0.99). The association between the higher number of communications and lower rates of infant HIV testing was only statistically significant in the intervention arm (adjusted OR: 0.91; 95% CI 0.84–0.98) but not in the control arm (adjusted OR: 0.97; 95% CI: 0.82–1.15).

## 4. Discussion

This study was designed to implement an SMS text message intervention to improve PMTCT service delivery by increasing communication between health workers and patients. The project was conducted in randomly selected government sites that are representative of facilities providing routine PMTCT services. In this study, we were unable to find a significant effect of SMS text messages on any of the key PMTCT milestones, including uptake and adherence to antiretroviral medications among mothers and infants, facility-based deliveries, or infant HIV testing at 6 weeks of age. However, very high uptake of antiretroviral medications and infant HIV testing was noted in both arms, much higher than that reported in program data from facilities within the region during the concurrent period [18]. Other studies within the region similarly did not identify such high usage of antiretrovirals. Washington et al. reported high antiretroviral dispensing in a cluster-randomized study that studied the effect of providing integrated HIV and antenatal care services in the same province in Kenya, but 39% of women given antiretrovirals did not actually start taking the medications [19]. Kinuthia et al. conducted a national survey in Kenya and reported 90% dispensing of antiretrovirals to mothers and infants for PMTCT along with high self-reported adherence to antiretrovirals but identified higher stigma indicators among the 10.6% of women who did not adhere to antiretrovirals during pregnancy or postpartum [20]. In our study, high antiretroviral dispensing during pregnancy was coupled with high reported adherence among women and their infants with the important biologic endpoint of very low transmission rates in both groups. Retraining of health workers on PMTCT may have led to improvements in program implementation. However, we believe the improvements were mediated through the increase in communication that was observed in both the intervention and control sites. Although women in control sites did not receive text messages, there was a dramatic increase in direct communication with their community health workers that could have contributed to the measured outcomes. Further analyses of participant and health care worker focus group discussions that were conducted during the course of our study are planned to elucidate whether the high rates of adherence we report in this study are related to a positive effect of increased communication, with or without the SMS messages, on health-seeking behavior.

We recorded high rates of infant testing in this study, with the additional finding of very low transmission rates of less than 2% at 6 weeks postpartum irrespective of allocation to intervention or control sites. Our infant HIV testing rates are significantly higher than those reported from national program data within the region during the corresponding period [21]. Washington et al. reported 6-week infant HIV testing in the range of 18–25% and infant HIV infection rates in the range of 4.2–6.6% in their control versus intervention sites within Nyanza province [19]. These data derived from similar time periods in the same region suggest that the increased communication achieved in our study may have led to the dramatic results in health-seeking behaviors and low HIV transmission rates.

Of particular interest is the even higher frequency of communication among health workers and caregivers who did not bring in their infants for HIV testing on schedule in the intervention arm. “We did not capture the reasons for communication to discern whether the uptake in communication was due to increased efforts by health workers to encourage infant testing or whether the communication originated from caregivers who were having difficulty bringing an infant into the facility for testing. Further analyses are planned to explore the nature of these communications based on focus group discussions that were conducted during the course of this study that may provide more insight regarding these seemingly disparate findings.”

Delivery in a health facility is associated with lower infant mortality [22]. We did not see any change in the proportion of women who delivered within a health facility despite increases in communication. However, communication alone is unlikely to mitigate previously identified deterrents to facility-based deliveries, security concerns with nighttime onset of labor, long distances, and cost [20]. Studies that have coupled use of mobile health communication with transport reimbursement in Uganda demonstrated a significant decrease in time to initiation of antiretroviral therapy among individuals with low CD4+ T-lymphocyte counts [23]. Our findings demonstrate the limitations of technology and communication, neither of which fully overcomes the structural barriers that women face in seeking facility-based deliveries. Further studies are warranted that address the structural and financial impediments to facility-based deliveries, in conjunction with enhanced communication.

This study was designed using what is perceived in the field to be the most rigorous study design, the cluster-randomized controlled approach, but several limitations are still apparent. We selected to randomize the level of the facility to mitigate the potential for the intervention to reach women not randomized to the intervention. Despite the facility-based randomization, increased communication was seen in both the intervention and control settings that may have blunted the potential effect of the intervention with the resulting null effect of the intervention compared to the control on PMTCT outcomes. Changes in comparator “control” arm effects during the design phase of studies and preceding study initiation remain a challenge in implementation research, where external factors such as evolving guidelines, improvements in health services delivery, and changes in knowledge and attitudes outside of interventions being implemented can affect study results. Novel study design and analytic methods are needed to accommodate the dramatic improvements in health services delivery being achieved in HIV prevention, care, and treatment.

Our data provide evidence for the potential role of increased communication, by text message, by phone calls, or in person, on effective PMTCT program implementation resulting in high antiretroviral usage during and low in utero and early postnatal HIV transmission in a rural area with limited resources. Our results support the importance of adequate training and empowerment of community health workers to improve communication with patients. The long-term effectiveness of improved communication to improve PMTCT services using mobile phones, not necessarily a prepopulated SMS texting platform with its associated costs, needs to be tested. Replication of such studies in other settings is critical to determine the generalizability of our findings and to identify optimal delivery strategies in varying settings, alongside cost and human resource considerations. In addition, further data are needed to determine the continued effect of such communication for prevention of postnatal HIV transmission during ongoing exposure through breastfeeding.

## Figures and Tables

**Table 1:** Study population baseline characteristics.

Variable	Overall	Intervention	Control	*p* value
	*N* = 550	*N* = 280	*N* = 270	
^*∗*^Age in years (IQR)	25.6 (22, 29)	25.5 (21, 29)	25.6 (22, 29)	0.784^1^
New HIV diagnosis in ANC	332 (60.4%)	155 (55.4%)	177 (65.6%)	0.015^2^
Yes				
Education level				
None	2 (0.4%)	1 (0%)	2 (0.7%)	0.021^2^
Primary	462 (84.0%)	247 (88.2%)	215 (79.6%)	
Secondary	77 (14.0%)	29 (10.4%)	48 (17.8%)	
College	9 (1.6%)	4 (1.4%)	5 (1.8%)	
Primiparous				
Yes	58 (10.6%)	32 (11.5%)	26 (9.7%)	0.493^2^
Number of children alive at enrollment (IQR)	2.7 (1.4)	2.8 (1.4)	2.6 (1.4)	0.212^3^
Gestational age at first ANC (IQR)	24 (20, 28)	24 (20, 28)	24 (20, 28)	0.173^1^
CD4+ T-lymphocyte count per cu mm, (IQR)	486 (349, 649)	444 (345, 541)	562 (480, 823)	0.003^1^
	(*n* = 57)	(*n* = 29)	(*n* = 86)	
CD4 count < 350	23/86 (26.7%)	18/57 (31.6%)	5/29 (17.2%)	0.201^2^
Hemoglobin g/L (SD)	105.7 (15.1)	104.9 (17.2)	106.4 (13.2)	0.486^3^
Time, in minutes, to health facility (IQR)	60 (30, 90)	60 (45, 120)	60 (30, 60)	<0.001^1^

^*∗*^Numbers are medians.

^1^Wilcoxon rank-sum test.

^2^Fisher's exact test.

^3^ *t*-test.

**Table 2:** Antiretroviral usage among study participants.

	Intervention	Control	Adjusted effect estimates	
			Difference [95% CI]^*∗*^	Relative risk [95% CI]
Antiretroviral usage outcomes at 34–36 weeks	*N* = 261	*N* = 242		
*Mother taking any type of antiretrovirals *	254 (97.3%)	241 (99.6%)	0.40% [−1.9, 2.7]	1.04 [0.91, 1.18]
*Mother on combination antiretroviral therapy *	51 (20.1%)	51 (21.2%)	−0.10% [−4.9, 4.7]	1.01 [0.29, 3.45]
*Any missed doses in the past week*	19 (7.5%)	13 (5.4%)	0.20% [−1.1, 1.6]	1.25 [0.43, 3.6]
Outcomes at delivery, 7 days postpartum	*N* = 247	*N* = 229		
*Mother taking any type of antiretrovirals *	234 (94.7%)	229 (100%)	−0.10% [−2.6, 2.3]	1.01 [0.88, 1.16]
*Mother on combination antiretroviral therapy *	59 (25.2%)	50 (21.8%)	0.70% [−4.3, 5.8]	1.28 [0.36, 4.62]
*Any maternal missed doses in past week*	18 (7.8%)	12 (5.3%)	0.40% [−1.3, 2.1]	1.56 [0.43, 5.61]
*Infant started taking antiretrovirals*	199 (80.9%)	209 (90.1%)	−1.20% [−4.8, 2.4]	0.91 [0.77, 1.14]
Outcomes 6–8 weeks postpartum	*N* = 244	*N* = 227		
*Mother taking any type of antiretrovirals*	178 (73%)	200 (88.1%)	−2.10% [−7.3, 3.1]	0.87 [0.61, 1.24]
*Mother on combination antiretroviral therapy *	49 (27.5%)	52 (26%)	−0.20% [−4.9, 4.6]	0.92 [0.28, 3.09]
*Any maternal missed doses in the past week*	19 (10.7%)	7 (3.5%)	0.80% [−1.1, 2.8]	2.09 [0.37, 11.77]

^*∗*^Based on cluster residuals.
